# Morphological and Proteomic Responses of *Eruca sativa* Exposed to Silver Nanoparticles or Silver Nitrate

**DOI:** 10.1371/journal.pone.0068752

**Published:** 2013-07-18

**Authors:** Candida Vannini, Guido Domingo, Elisabetta Onelli, Bhakti Prinsi, Milena Marsoni, Luca Espen, Marcella Bracale

**Affiliations:** 1 Dipartimento Biotecnologie e Scienze della Vita, Università degli Studi dell’ Insubria, Varese, Italy; 2 Dipartimento Bioscienze, Università degli Studi di Milano, Milano, Italy; 3 Dipartimento di Scienze Agrarie e Ambientali Produzione, Territorio, Agroenergia, Università degli Studi di Milano, Milano, Italy; Institute of Botany, Chinese Academy of Sciences, China

## Abstract

Silver nanoparticles (AgNPs) are widely used in commercial products, and there are growing concerns about their impact on the environment. Information about the molecular interaction of AgNPs with plants is lacking. To increase our understanding of the mechanisms involved in plant responses to AgNPs and to differentiate between particle specific and ionic silver effects we determined the morphological and proteomic changes induced in *Eruca sativa* (commonly called rocket) in response to AgNPs or AgNO_3_. Seedlings were treated for 5 days with different concentrations of AgNPs or AgNO_3_. A similar increase in root elongation was observed when seedlings were exposed to 10 mg Ag L^1^ of either PVP-AgNPs or AgNO_3_. At this concentration we performed electron microscopy investigations and 2-dimensional electrophoresis (2DE) proteomic profiling. The low level of overlap of differentially expressed proteins indicates that AgNPs and AgNO_3_ cause different plant responses. Both Ag treatments cause changes in proteins involved in the redox regulation and in the sulfur metabolism. These responses could play an important role to maintain cellular homeostasis. Only the AgNP exposure cause the alteration of some proteins related to the endoplasmic reticulum and vacuole indicating these two organelles as targets of the AgNPs action. These data add further evidences that the effects of AgNPs are not simply due to the release of Ag ions.

## Introduction

At the nanometer scale, many materials possess unique electrical, chemical, and physical properties that are exploited in electronics, medicine, energy production, healthcare and environmental remediation. Due to the increase in the production of synthetic nanoparticles (NPs), their potential release into the environment is estimated to increase dramatically in the coming years. For this reason, nanoecotoxicology is an emerging field of research [1]. To ensure sustainable use of nanomaterials, their fate and impact on the environment should be understood.

The interactions of nanomaterials with plants have not been fully elucidate. There are different and often conflicting reports on the absorption, translocation, accumulation, biotransformation, and toxicity of NPs on various plant species. Many questions remain concerning the fate and interactions of NPs in plant cells [2]. A few studies of NPs have been performed on food crops; however, their possible effects in the food chain are unknown [3].

Because of their antimicrobial properties, silver nanoparticles (AgNPs) are among the most widely used species of nanoparticles in commercial products including textiles, plastics, paints, personal care products, and food storage containers [4].

Toxicological studies of AgNPs have been conducted on bacteria, animal cells, and algae [5]–[7]. The impact of AgNPs on higher plants appears to depend on the species and age of plants, the size and concentration of the particles, the experimental conditions such as temperature, and the duration and method of exposure. For example, 10 mg L^−1^ AgNPs reduces seed germination in *Hordeum vulgare* and shoot length in *Linum usitatissimum* and *Hordeum vulgare* [8]. However, 100 mg L^−1^ AgNPs has no significant effect on seed germination in *Cucumis sativus*, and *Lactuca sativa* [9]. Other studies indicate a positive role for AgNPs in the promotion of plant growth in *Brassica juncea* [10], *Panicum virgatum,* and *Phytolacca americana* [11], *Phaseolus vulgaris,* and *Zea mays* [12].

The molecular mechanisms that mediate the effects of AgNPs in plants remain unknown. It is important to increase our knowledge of these mechanisms before implementing a large-scale agricultural utilization of AgNPs as vehicles for the delivery of pesticides and herbicides.

In this paper, we performed a meso-analysis of the response of *Eruca sativa* (rocket), a fast-growing crop commonly used in phytotoxicity tests, to AgNPs and AgNO_3_. The analyses included dose-response tests, cellular and ultrastructural microscopy, and proteomics. Although the proteomic approaches have extensively contributed to understand plant heavy metal response [13], to the best of our knowledge, they never have been applied to study NPs effects in plants. A few proteomic studies have been performed to analyze the effects of NPs in animal cells and bacteria [14]–[17]. We compared the proteomic profiles that resulted from plant exposure to AgNPs or AgNO_3_ (added at equivalent Ag concentration). The data indicated that different patterns of plant responses arose from exposure to the different Ag compounds and highlight previously uncharacterized cellular phenomena related to the interaction of AgNPs with plant cells. The outcome of this work may be useful to determine the biocompatibility of AgNPs and to identify potential agricultural applications for nanoparticles.

## Materials and Methods

### Materials

All experiments were carried out using commercially manufactured 10 nm AgNPs coated with Poly Vinyl Pyrrolidone (PVP) to avoid NP aggregation (Biopure AG10, Nanocomposix, San Diego, CA). All experimental concentrations were prepared by diluting the AgNP stock solution (1 g L^−1^) in deionised water. Also silver nitrate (AgNO_3,_ Sigma-Aldrich) was dissolved in deionised water. 50 mg L^−1^ of cysteine (Sigma-Aldrich) was added alone (control) or in combination with 10 mg Ag L^−1^ of either AgNPs or AgNO_3_. All dilutions were freshly prepared before use.

### Nanoparticle Characterization

The shape and the size of PVP-AgNPs were determined by Transmission Electron Microscopy (TEM): a drop of 10 mg L^−1^ AgNPs was placed on formvar/carbon coated nickel grids and dried in air. Grids were examined by an EFTEM LEO 912AB transmission electron microscope (Zeiss) working at 80 kV. Diameter of PVP-AgNPs was measured by Esivision software and average and standard deviation were calculated. Ag concentration in AgNP suspensions was determined by flame atomic absorption spectroscopy (F-AAS; Thermo-Electron Atomic Absorption Spectrometer) after addition of 1% HCl as described below.

### Seed Treatment

Seeds of *Eruca sativa* Mill. (Franchi Sementi, Milan, Italy) were surface sterilised with 10% sodium hypochloride solution for 10 min and then rinsed with distilled water. For each treatment, 200 seeds were soaked for 4 h in 5 ml of either 0.1, 1, 10, 20, 100 mg L^−1^ Ag of either PVP-AgNPs or AgNO_3_. We examined the coating effects by exposing additional replicates to 0.1, 1, 10 mg L^−1^ of PVP. Deionised water was used as control. A filter paper moistened with 4 ml of test solution was put into each 100 × 15 mm sterilized Petri dish. Seeds were transferred onto the filter paper with twenty five seeds per dish. Each concentration point of the treatments was performed five times. All treatments were conducted in triplicate. Dishes were placed for 5 days in the dark under controlled temperature (25±1°C). At the end of the exposure, seedlings were washed three times with 0.1.M EDTA and then with MilliQ-water. Roots and shoots were measured with a ruler, separated and immediately frozen at −80°C.

### Plant Ag Content Determination

Shoots and roots frozen samples, were lyophilized and then dried in an oven at 60°C for 8 h. Dried shoots and roots samples were treated with 1 mL of HCl and 1 mL of hydrogen peroxide (H_2_O_2_) and reduced to dryness on a hotplate. Samples were reconstituted in 1% HNO_3_ in Milli-Q water. Blanks were made with the same solvents and chemicals employed in the treatment and digestion of the samples, or with just 1% HNO_3_ in Milli-Q water. All the chemicals used for the sample pre-treatments and mineralization were for metal trace analysis (or equivalent) grade: Milli-Q water (Millipore purification system); hydrochloric acid (Baker 9530 for metal trace analysis, 36.5–38%); nitric acid (Baker 9598 for metal trace analysis, 69–70%); and H_2_O_2_ (Fluka 95313, not stabilized, 30%). Calibration standard solutions were prepared from 1000 mgLl^−1^ standard solutions of Ag (Baker Instra-Analyzed).

### Light and Transmission Electron Microscopy (LM and TEM)

2 mm root samples were fixed in 4% formaldehyde and 2% glutaraldehyde in cacodylate buffer 0.1 M pH 6.9 After three days, the samples were rinsed in 0.1 M cacodylate buffer and post-fixed in 1% osmium tetroxide in cacodylate buffer pH 6.9 for 2 h at 4°C. Samples were then dehydrated with increasing concentrations of ethanol and embedded in Spurr resin. Thin section (2 µm) and ultra-thin sections (80 nm) were obtained using a Reichert Jung Ultracut E microtome. For optical microscopy section were stained with toluidin blue and observed with an DMRB Leica microscopy. For electron microscopy sections were stained with 3% uranyl-acetate and lead citrate and observed with an EFTEM LEO 912AB transmission electron microscope (Zeiss) working at 80 kV. AgNPs were enhanced with QH silver (Nanoprobes) for 4 minutes as described by the manufacturer. Five plants were analyzed for each type of treatment. All treatments were conducted in triplicate.

### Protein Sample Preparation and Two-dimensional IEF/SDS–PAGE

Frozen roots were homogenized by using mortar and pestle in liquid nitrogen with an addition of sand quartz. Total proteins were extracted and their concentration measured as previously described [18].The samples were directly loaded for isoelectrofocusing (IEF) or stored in aliquots at −80°C until use. Three independent experiments and extractions for each experimental condition were performed.

IEF was carried out with 700 µg of total protein extract by using an immobilized 4–7 pH gradient (Immobiline DryStrip, 18 cm; Amersham Biosciences, Uppsala, Sweden). Two dimensional electrophoresis were performed as previously described [18].Proteins were detected with colloidal Coomassie brilliant blue (CBB) modified [19]. Three gel replicas were performed.

The gels were scanned at a high resolution (300 dpi, 16-bit greyscale pixel depth) with a GS-800 densitometer (Bio-Rad). Image analysis and statistical calculations were performed using the Progenesis SameSpots software (NonLinear Dynamics, Newcastle, UK). All sample gel images were aligned, and then spots were automatically detected and filtered to eliminate non-protein spots. Only the spots with a fold change of ±1.5 and ANOVA p-value ≤0.05 were accepted as differentially expressed, excised from the gel and digested for LC-MS/MS analysis [19].

### Liquid Chromatography-ElectroSpray Ionization-tandem Mass Spectrometry (nanoLC-nESI-MS/MS)

The tryptic peptides were analyzed on an Agilent 6520 Q-TOF with an HPLC Chip Cube source (Agilent Technologies). Peptides were separated by a C18 column using an acetonitrile gradient (from 5% to 60% v/v) in 0.1% (v/v) formic acid at 0.4 µL min^−1^. The analyses were conducted in auto-MS/MS positive mode with an active exclusion of 2 spectra for 0.1 min. Spectra were interpreted by Spectrum Mill MS Proteomics Workbench. Carbamidomethylation of cysteines and oxidation of methionines were set as fixed and variable modifications, respectively, accepting 2 missed cleavages *per* peptide. The search was conducted against the subset of *Brassicaceae* protein sequences downloaded from the National Center for Biotechnology Information (NCBI, http://www.ncbi.nlm.nih.gov.) The database was concatenated with the reverse one. The threshold used for peptide identification was Spectrum Mill score ≥9, SPI% ≥50% and the difference between forward and reverse scores ≥2. If needed, protein similarity search was performed against the NCBI-nr database using the FASTS algorithm (http://fasta.bioch.virginia.edu/fasta_www2/fasta_list2.shtml) [20].

Detailed information about instrumentation, analytical procedures and spectra interpretation is available in “Methods S1”.

### Semiquantitative RT-PCR Experiments

Total RNA was isolated using a Sigma Spectrum Plant Total RNA kit according to the manufacturer’s instructions. RNA concentration and quality were determined with a spectrophotometer. Samples were treated with DNase-I (Ambion). Total RNA (2 µg) was retro-transcribed using the Enhanced Avian RT First Strand Synthesis Kit according to manufacturer’s instructions (Sigma-Aldrich). 18S rRNA was used as the internal control.

The sequences of primers used were as follow: **JAC**: sense, 5′AGGAGAGGGTCCAGGGCCAA3′, antisense, 5′CCGGGGTCGAACACTGCAC3′. **MS**: sense,5′ACGCATCCCACAAGGCGGTG3′, antisense, 5′AGGCGCCAGTAAAGGCCTGG3′. **PRX**: sense, 5′ CACCACGGAGCTTGGTGCGA3′, antisense, 5′ TCTGTGCGACGCAGATAGCCT3′.

**SOD**: 5′CAACGCTGCTCAGGCGTGGA3′, antisense, 5′ GGCGGCTCCAAGTCTGGCAC3′. **MLP**: sense, 5′CAAGGTGTCACCATCCACGA3′ antisense, 5′CTGCTCCATCACGTGACCTT3′. **18S rRNA**: sense, 5′ TCCCGACCAGGGATCAGCGG3′ antisense, 5′AGCAGGCTGAGGTCTCGTTCGT3′. The conditions used for the PCR were as follows: 94°C for 5 min, 37 cycles of 94°C for 45 sec, 58°C for 45 sec and 72°C for 1 min, with final extension at 72°C for 5 min. Only for 18S rRNA the annealing temperature was 59°C and the PCR cycles were 25. PCR products were visualized by 1% agarose gel electrophoresis. The intensity of the bands was quantified using ImageJ 1.41 (http://rsb.info.nih.gov/ij/). All experiments were repeated three times.

### Statistical Analysis

All results were presented as mean of the replicates ± standard deviations (SD). Differences between treatments for the different measured variables were tested by one-way variance (Anova), followed by Tukey’s HSD post-hoc test when significant differences were found (p≤0.05).

## Results and Discussion

### AgNPs Characterization and their Effects on *Eruca sativa*

The size and shape of AgNPs were determined by TEM analysis (Figure S1). The mean size of the AgNPs calculated from TEM images was 14±0.3 nm (n = 402) with 77% of the particles ranging from 5–17.5 nm. Data from F-AAS of the experimental working dilutions of AgNp showed that the concentration of Ag in the stock suspension was 1 mg mL^−1^, which consistent with that reported by the manufacturer.

**Figure 1:**
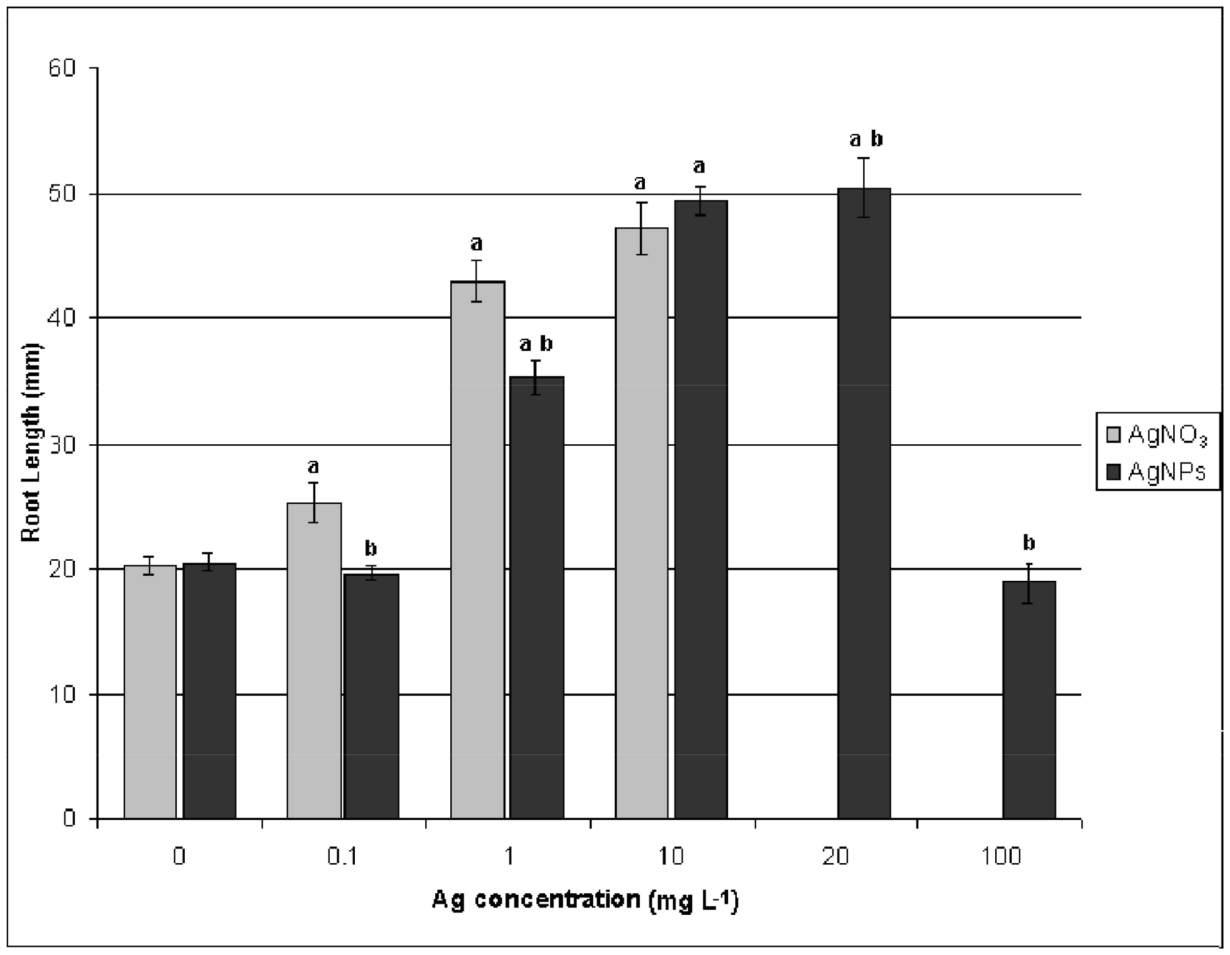
Concentration effects of PVP-AgNPs and AgNO3 on the root elongation of *Eruca sativa* after five days of exposure. ^a^Significantly different from the control (p<0.05) and ^b^ significantly different from the AgNO_3_ treatment (p<0.05).

**Figure 2:**
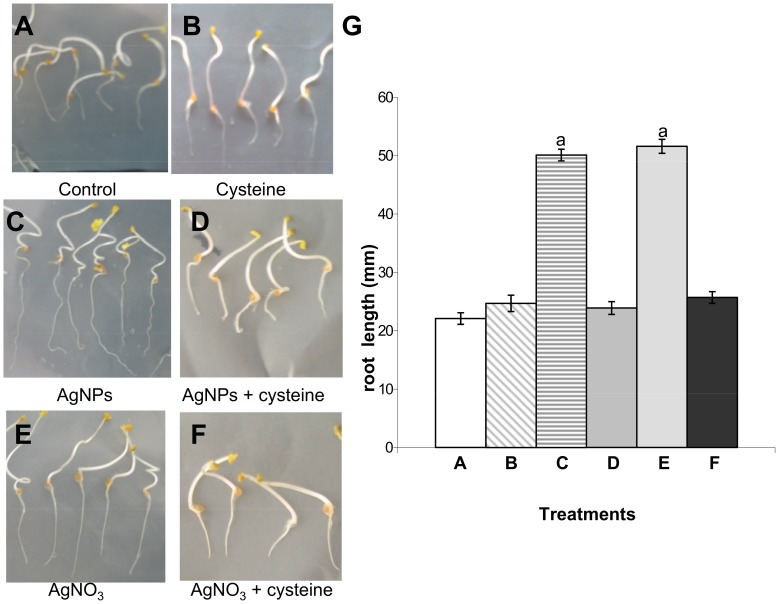
Effects of 10 mg Ag L^−1^ of either PVP-AgNPs or AgNO_3_ on the root growth of *Eruca sativa* and rescue by cysteine. G: results are shown as average ± SE of measurements of 400 seedlings per each condition. ^a^ Significantly different from the control (p<0.05).

For the dose-response studies with of PVP-coated AgNPs, concentrations of 0, 0.1, 1, 10, 20, and 100 mg L^−1^ were used. We tested the dosage effects on germination of *Eruca sativa* seeds and on elongation growth of roots and shoots. These are rapid tests that are already used to assess the phytotoxicity of NPs on different plant species [11], [21], [22]. Germination of control samples was greater than 90%; AgNP treatments did not show any significant effect on the percentage or rate of germination (data not shown). However, as shown in Figures 1 and 2C, AgNPs significantly stimulated radical growth. The maximum stimulation occurred at a concentration of 10–20 mg L^−1^ AgNPs. The root length of plants treated with 100 mg L^−1^ of AgNP was similar to that of the control sample (Figure 1). PVP treatments have no significant effect on germination or root and shoot elongation (data not shown). Our results confirm that AgNPs can lead to an improvement in plant growth, and are consistent with previously published data [10], [11], [12].

We also treated *Eruca sativa* seeds with AgNO_3_. As shown in Figures 1 and 2E, AgNO_3_ stimulated root growth up to the concentration of 10 mg Ag L^−1^, which is similar to the result obtained from treatment with AgNPs. However, increasing the AgNO_3_ concentration up to 20 mg Ag L^−1^ completely blocked germination. No statistically significant effects of AgNPs or AgNO_3_ treatments were observed on shoot elongation (data not shown). Five days of exposure of plant roots to either AgNPs and AgNO_3_ at a concentration of 10 mg Ag L^−1^ induced the same effect on root elongation. At this concentration, the Ag content of roots and shoots was not significantly different for either AgNPs or AgNO_3_ (Table S1). Most Ag was associated with the roots. The translocation factor for Ag concentration in shoots versus Ag concentration in roots, was 0.0017 and 0.0012 for AgNPs and AgNO_3_, respectively. This could explain why Ag treatments have a significant effect only on root elongation and not on shoot elongation.

To understand if the root elongation observed in *Eruca sativa* treated with AgNPs was due to the Ag^+^ released from NPs or to NPs themselves, 50 mg L^−1^ of cysteine was added to both Ag treatments. Cysteine is a strong silver ligand, proved to be useful in examining the contribution of dissolved Ag to the overall toxicity of AgNPs. The cysteine completely rescued root elongation in both treatments (Figure 2 D, F) showing evidence that the root elongation induced by AgNPs is mediated by Ag^+^.

We selected the concentration of 10 mg Ag L^−1^ of either AgNPs or AgNO_3_ to investigate the morphological and proteomic effects on *Eruca sativa* roots.

### Light and TEM Microscopy

The effects on *Eruca sativa* roots of treatment with either AgNPs or AgNO_3_ were examined using light microscopy and TEM. Light microscopy observations showed that most of roots treated with AgNPs had shorter root hairs or no root hairs compared to that of the control (data not shown). The cells of the root tip were more vacuolated in roots treated with AgNPs compared to those of the control (Figure S2). These effects were less severe in samples treated with AgNO_3_, and cells appeared more similar to the control (Figure S2). These results are consistent with those reported by Yin et al. [22] for *Lolium multiflorum* seedlings treated with 40 mg L^−1^ GA-coated AgNPs and AgNO_3_.

**Figure 3:**
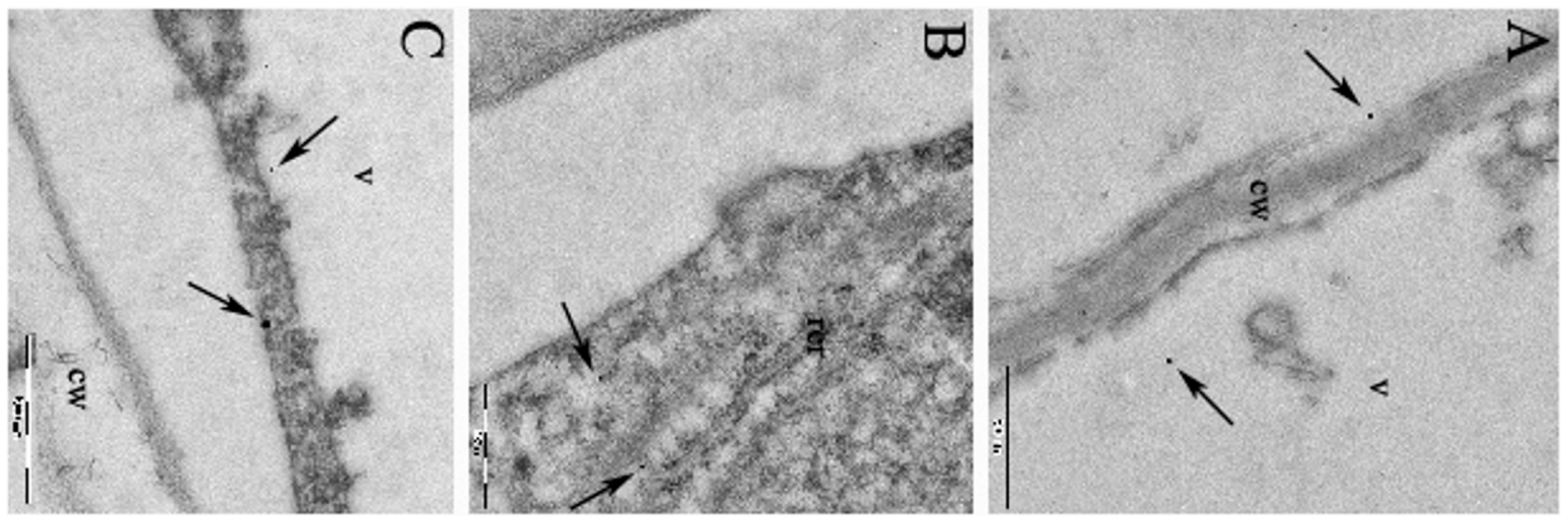
Transmission-electron micrographs of the roots of *Eruca sativa* exposed to 10 mgL^–1^ of 10 nm AgNP. In A, B, C, the arrows indicate the location of the small dark deposits in the meristematiccells. v, vacuole; cw, cell wall, rer, rough endoplasmic reticulum. Magnification bar: 500 nm.

Differences among the treatments were observed using TEM analysis of cell ultrastructure. In root cup columella cells treated with AgNPs or AgNO_3_, the main effect observed by TEM was a reduction in the number of the amyloplasts and the size of the smooth endoplasmic reticulum (SER). In particular, the SER disappeared almost completely in treated samples (Figure S2-D, H). Cysteine reverted the phenotypes in both cases (Figure S2-F, J). AgNPs also induce morphological modifications of SER in the region of cell elongation and differentiation of root samples. The SER appeared to be well extended in the cytoplasm of control cells (Figure S3-A), and sometimes it displayed branching or expansion in cisternae (Figure S3-C, D). AgNPs (but not AgNO_3_) induced pronounced morphological modifications of SER; in particular, an extensive swelling was observed (Figure S3-E). AgNPs have never been observed in root tissues by TEM analysis, which suggests that they remain on the root surface. The enhancement procedure put in evidence small, dark deposits in cells exposed to AgNPs, which probably originated from the nanoparticles absorbed onto the root surface (Figure 3). These deposits were never observed in control roots. This could indicate that the effects of AgNPs observed in our experimental system are mediated primarily by Ag^+^ released by oxidative dissolution of NPs at the root interface in the presence of secreted root metabolites. Kim et al. [23] reached a similar conclusions from their work on the phytotoxicity of CuO and ZnO NPs in *Cucumis sativus.* However, the small dots observed within the cells may contribute Ag by releasing the ions locally in the vicinity of the cellular compartments or molecular targets.

### 2-DE Analysis

**Figure 4:**
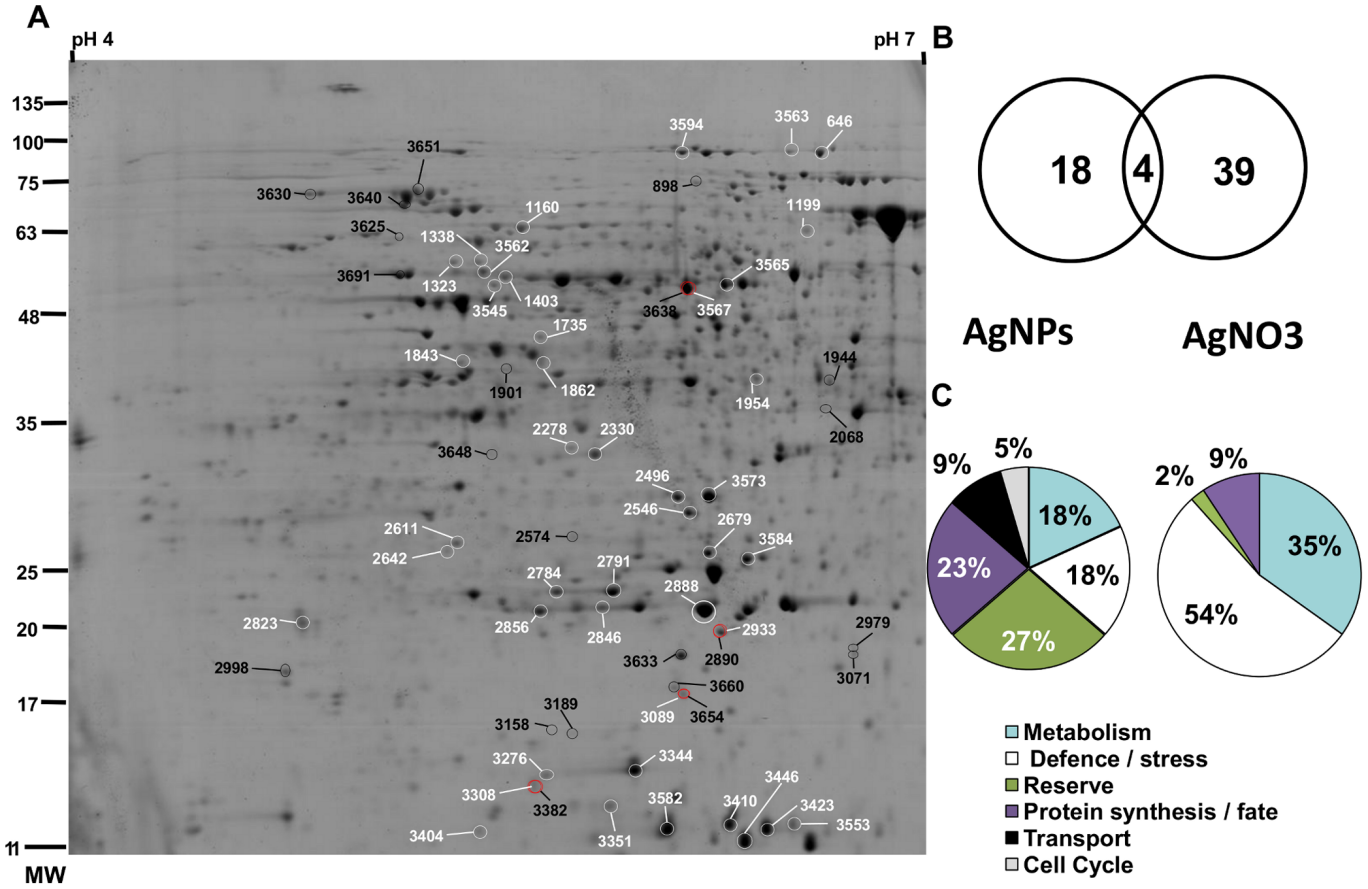
Two-dimensional electrophoresis. A) The differentially expressed protein spots in samples treated with AgNPs (dark labels) or with AgNO_3_ (white labels) with respect to the control are marked on a representative 2-DE gel. The numbers correspond to the spots numbers listed in Tables S2 and S3. The spots that changed in both treatments are red labeled. B) Venn diagram showing the degree of overlap between significantly regulated proteins from the treatment with 10 mg Ag L^−1^ of either PVP-AgNPs or AgNO_3_. C) Functional classification of proteins that change significantly in relative abundance in AgNP- orAgNO_3_- treated samples with respect to the control.

Proteomic analysis of AgNPs and AgNO_3_ was conducted at the Ag concentration that induces the same root elongation, which was determined to be 10 mg Ag L^−1^. For both treatments, approximately 1000 spots were resolved and detected by colloidal CBB staining over a pH range of 4–7 and a size range of 10–250 kDa (Figure 4A). All spots were matched by gel-to-gel comparisons. Differences in the relative abundance (Vol%) of each spot were evaluated by software-assisted analysis. The ANOVA test (*p*<0.05), coupled with a threshold of 1.5-fold change in level, revealed 22 and 43 differentially expressed protein spots in samples treated with AgNP or AgNO_3_, respectively, compared to that of the control. The differentially expressed protein spots are marked on the representative 2-DE gels shown in Figure 4A. In AgNP-treated samples, seven protein spots were down-regulated (32%), wheres only four spots decreased in AgNO_3_-treated samples (9%). The Venn diagram in Figure 4B, shows that only the levels of four proteins that changed were common to both treatments, whereas 18 and 39 proteins were specifically expressed following AgNP or AgNO_3_ treatments, respectively. The low level of overlap of differentially expressed proteins indicates that AgNPs and AgNO_3_ cause distinct changes in the proteome of the root cells even though some common features persist (see below). Studies on other organisms [6], [24], [25] have also revealed differences in the gene expression patterns of cells exposed to NPs or to the corresponding free ions.

Excised spots were in-gel digested and analyzed by nLC–nESI-MS/MS. The identified proteins were classified into different functional categories according to their putative physiological functions (Figure 4C, Tables S2, S3 and S4).

**Figure 5:**
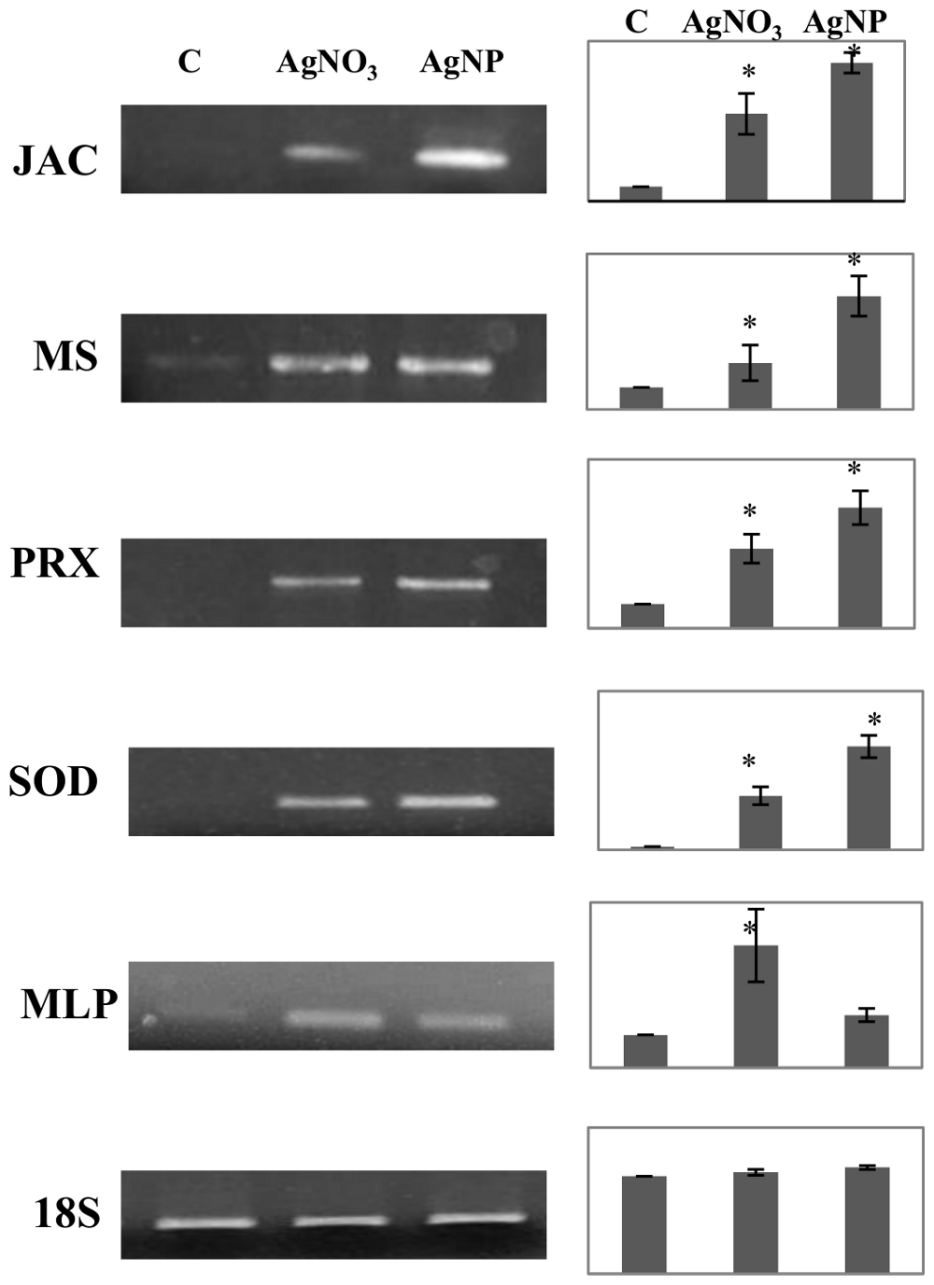
RT- PCR espression analysis of selected genes that displayed differential accumulation on 2D protein maps. Total RNA was extracted from non treated plants (C) and samples treated with AgNO_3_ and AgNPs. 18S rRNA, which displays constitutive expression in all samples, was used as internal control. * indicates values that are significantly different from control with p<0,05.

To validate the proteomic data we performed semiquantitative RT-PCR analysis. Candidate proteins were selected as representatives of different functional categories. (Figure 5).

Investigation of the differentially expressed proteins revealed insights into the mode of action of AgNPs or AgNO_3_. AgNP and Ag ions in the form of AgNO_3_ appear to share some common mechanisms of action. Both treatments induce the accumulation of proteins related to sulfur metabolism. In particular, AgNPs and AgNO_3_ strongly induce proteins of the jacalin lectin family (JAC). These seed-specific proteins catalyze the hydrolysis of glucosinolates (sulfur-containing secondary metabolites present in cruciferous plants) and release nitrile and sulfate [26]. The nitrile has auxin activity and can be converted to indole-3acetic acid [27]. This fact may explain (at least in part) the root growth stimulation observed in seedlings germinated in the presence of AgNPs and AgNO_3_. The root elongation is induced by both Ag treatments indicating that this effect is related to the Ag ions. This hypothesis is consistent with the observed rescue of root elongation by cysteine addition.

In Cruciferae, glucosinolate hydrolysis can be a source of sulfate when necessary. For example, during sulfur deficiency, the transcription of jacalins is induced in Arabidopsis [28]. A number of molecular studies revealed the involvement of sulfur metabolism in metal stress tolerance in plants [29]. Sulfur is an important constituent of many stress-related compounds, such as glutathione (GSH), cysteine, methionine and thioredoxin. Both Ag treatments consistently induced two key enzymes in cysteine biosynthesis: O-acetylserine(thiol)lyase in AgNO_3_-treated roots, and cysteine synthase in AgNPs-treated roots. Cysteine can chelate dissolved Ag ions and alter the surface chemistry, aggregation, and dissolution of zero-valent silver nanoparticles [30]. Moreover, cysteine is a direct coupling step between sulfur and its incorporation into GSH, a key player in plant stress tolerance to radical oxygen species (ROS). In addition, AgNP exposure cause the accumulation of a vitamin-B12-independent methionine synthase isozyme (MS) involved in the biosynthesis of the methionine, the second principal sulfur-containing amino acid. As shown in Figure 5, the significant increases at the protein level were confirmed at the mRNA level for JAC and MS. These results indicate that in both Ag treatments the metabolism of sulfur not only plays an important role in growth and development of roots but it is also involved in Ag tolerance.

Both Ag treatments activate some common enzymatic and non-enzymatic pathways of ROS detoxification machinery, including superoxide dismutase (SOD) and Type2 peroxiredoxin (PRX). The SOD enzyme is a component of the first line of cellular defense against oxidative stress and functions in early scavenging of superoxide radicals and converting them to hydrogen peroxide. Increased expressions of SOD isoforms have been documented in plant exposed to excess metals [13]. PRX is a thiol peroxidase with multiple functions; it detoxifies hydroperoxides and is a redox sensor.

Although these proteins were affected similarly by both treatments, there also were several differences. Only the treatment with AgNPs induced the accumulation of the glyoxalase I enzyme detoxificant methylglyoxale, which is a cytotoxic by-product of glycolysis that accumulates in cells in response to environmental stresses [13]. Treatment with AgNO_3_ increased the abundance of several major latex proteins, four glutathione-S-transferases,, an isoflavone reductase and a universal stress protein. These proteins are leitmotifs in metal detoxification [31].

In addition to these proteins, we found three AgNO_3_-responsive proteins with a less well known role in the metal response: the class I glutamine amidotransferase, the tryptophan synthase alpha chain and the metacaspase-4 subunit p. Transgenic Arabidopsis plants with elevated levels of class I glutamine amidotransferase have increased stress protection against environmental stress conditions through cytosolic SOD activation [32]. The overexpression of the tryptophan synthase beta chain and the increased level of tryptophan in *Arabidopsis* plants reduce lipid peroxidation and enhance the tolerance to Cd [33]. Arabidopsis metacaspase 2d is a positive mediator of cell death induced during biotic and abiotic stresses [34]. The accumulation of the first two proteins and the decrease of the third suggest that these enzymes play an important role in AgNO_3_ tolerance.

All these data show that in our experimental system: 1) AgNPs cause oxidative stress, as also confirmed by the higher level of mRNA expression for SOD and PRX obtained using semiquantitative RT-PCR analysis (Figure 5). Similar results were reported in *Lemna gibba, Brassica juncea, Caenorhabditis elegans,* zebrafish, Drosophila larvae and mammalian cells [35]–[37]; 2) AgNPs and AgNO_3_ activate common and specific elements for effective detoxification. It will be of interest to investigate if this response to AgNPs is common to other plants.

The exposure to AgNP is able to alter specific cellular functions. In fact, AgNPs change the expression of some proteins related to protein folding. In particular, we found the down-regulation of two chaperones: the ER-resident luminal-binding protein 1 (BiP1) and the heat shock protein 70–2, involved in ER-associate degradation. These data show for the first time in plants that AgNPs induce perturbations in the functions of the ER, which is already known for mammalian cells [38]–[40]. In addition, in AgNPs-treated roots the up-regulation of the beta-glucosidase 23 (PYK10), which is a major component in the ER body, confirms that the ER might be a target of the AgNps. The involvement of ER in AgNP stress is consistent with the observed ER morphological alterations.

A second important response found only in roots treated with AgNPs is the decrease of two vacuolar-type proton ATPase (V-ATPase) subunits. V-ATPase plays a central role in vacuole acidification energizing the active transport of ions across the tonoplast. Moreover, it plays an important role in the trans-Golgi network. Morphological data showed that five days of exposure to 10 mg Ag L^−1^ of either AgNPs or AgNO_3_ produced more cytoplasmic vacuolization in root tip cells. The vacuole is the main place to store toxic compounds in plants. Nevertheless the down-regulation of two V-ATPase subunits could indicate greater toxicity of AgNPs.

The exposure to AgNO_3_ caused a specific increase of several proteins involved in energy production including plastidial and mitochondrial ATP synthase subunits, carbonic anhydrase, and aconitate hydratase. This up-regulation might help cells to produce more reducing power to facilitate the response to AgNO_3_ stress.

In conclusion, we provided a representative proteome-wide map of the effects induced by AgNPs and AgNO_3_ in *Eruca sativa* roots. It is difficult to extrapolate the extent to which metal ions released from the NPs caused the changes we observed with the AgNPs. The results show that, although the macroscopic cell response to the two treatments is similar, the effects from the two treatments only partially overlap at a molecular level. The data from the proteomic studies strongly indicate that the effects of AgNPs are not due solely to the release of Ag^+^ into the surrounding environment. To the best of our knowledge, we identified for the first time several players involved in different pathways during the response to stress induced by AgNPs.

## Supporting Information

Figure S1 **Detailed characterization of AgNPs.** A: representative TEM image of 10 mg L^−1^ AgNp suspension. Magnification bar = 100 nm. B and C: size distribution of AgNPs.
(TIF)

Figure S2 **Light microscope and TEM observations.** *Eruca sativa* primary root tips after 5 days of exposure to water control (A and B); 10 mg Ag L^−1^ of either AgNPs (C and D) or AgNO_3_ (G and H); 50 mg L^−1^ cysteine +10 mg Ag L^−1^ of either AgNPs (E and F) or AgNO_3_ (I and J). In A, C, E, G, I the magnification is 54x and the arrows point to the columella cells. In B, D, F, H, J the arrows indicate the Smooth Endoplasmic Reticulum (SER). a, amiloplast; v, vacuole; n, nucleus and m, mitochondrion. Magnification bar: B and D = 2 µm; F–J = 1 µm.
(TIF)

Figure S3 **TEM photographs showing the region of cell differentiation.** Root cells exposed to water (A, B, C and D); 10 mg Ag L^−1^ of either AgNPs (E) or AgNO_3_ (G); 50 mg L^−1^ cysteine +10 mg Ag L^−1^ of either AgNPs (F) or AgNO_3_ (H and I). Arrows point to the Smooth Endoplasmic Reticulum (SER). v, vacuole; m, mitochondrion; n, nucleus, cw, cell wall; pp, proplastid. Magnification bar: A = 2 µm; B and C = 500 nm; D–I = 1 µm.
(TIF)

Table S1 **Measurement of Ag content by F-AAS.**
(DOC)

Table S2 **Differentially expressed proteins in samples treated with AgNPs with respect to the control identified by CHIP-q-TOF MS/MS analysis.**
(DOC)

Table S3 **Differentially espressed proteins in samples treated with AgNO_3_ with respect to the control identified by CHIP-q-TOF MS/MS analysis.**
(DOC)

Table S4 **Statistical data about protein identification by nanoLC-nESI-MS/MS analysis.** Statistical data about protein identification by CHIP-q-TOF analysis coupled with spectra interpretation by “Spectrum Mill MS Proteomics Workbench Rev A.03.03.084 SR4” (Agilent technologies).
(XLS)

Methods S1 **NanoLiquid Chromatography-nanoElectroSpray Ionization-tandem mass spectrometry (nLC-nESI-MS/MS).**
(DOC)

## References

[1] Muller NC, Nowack B (2008) Exposure modeling of engineered nanoparticles in the environment. Environ Sci Technol 42: 4447–4453. 18605569 10.1021/es7029637

[2] Ma X, Geiser-Lee J, DengY, Kolmakov A (2010) Interactions between engineered nanoparticles (ENPs) and plants: Phytotoxicity, uptake and accumulation. Sci Total Env 408: 3053–3061. 20435342 10.1016/j.scitotenv.2010.03.031

[3] Rico CM, Majumdar S, Duarte-Gardea M, Peralta-Videa JR, Gardea-Torresdey L (2011) Interaction of nanoparticles with edible plants and their possible implications in the food chain. J Agric Food Chem. 59: 3485–3498. 10.1021/jf104517j PMC3086136 21405020

[4] An inventory of nanotechnology-based consumer products currently on the market. Available: http://www.nanotechproject.org/inventories/consumer/analysis_draft/. Accessed 2013 Jun 10.

[5] Fabrega J, Luoma SN, Tyler CR, Galloway TS, Lead JR (2011) Silver nanoparticles: Behaviour and effects in the aquatic environment. Environ Int 3: 517–531. 10.1016/j.envint.2010.10.012 21159383

[6] Poynton HC, Lazorchak JM, Impellitteri CA, Blalock BJ, Rogers K, et al (2012) Toxicogenomic Responses of Nanotoxicity in *Daphnia magna* Exposed to Silver Nitrate and Coated Silver Nanoparticles. Environ Sci Technol 46: 6288–6296. 22545559 10.1021/es3001618

[7] Navarro E, Piccapietra F, Wagner B, Marconi F, Kaegi R, et al (2008) Toxicity of silver nanoparticles to *Chlamydomonas reinhardtii.* . Environ Sci Technol 42: 8959–8964. 19192825 10.1021/es801785m

[8] El-Temsah YS, Joner EJ (2010) Impact of Fe and Ag nanoparticles on seed germination and differences in bioavailability during exposure in aqueous suspension and soil. Environ Toxicol 27: 42–49. 20549639 10.1002/tox.20610

[9] Barrena R, Casals E, Colon J, Font X, Sanchez A, et al (2009) Evaluation of the ecotoxicity of model nanoparticles. Chemosphere 75: 850–857. 19264345 10.1016/j.chemosphere.2009.01.078

[10] Sharma P, Bhatt D, Zaidi MG, Saradhi PP, Khanna PK, et al (2012) Silver nanoparticle-mediated enhancement in growth and antioxidant status of *Brassica juncea* . Appl Biochem Biotechnol 167: 2225–33. 22692847 10.1007/s12010-012-9759-8

[11] Yin L, Colman BP, McGill BM, Wright JP, Bernhardt ES (2012) Effects of Silver Nanoparticle Exposure on Germination and Early Growth of Eleven Wetland Plants. PLoS ONE 7(10): e47674 doi:10.1371/journal.pone.0047674 23091638 10.1371/journal.pone.0047674 PMC3473015

[12] Salama HMH (2012) Effects of silver nanoparticles in some crop plants, Common bean (*Phaseolus vulgaris* L.) and corn (*Zea mays* L.). Int Res J Biotech 3: 190–197.

[13] Hossein Z, Komatsu S (2013) Contribution of proteomic studies towards understanding plant heavy metal stress response. Frontiers Plant Sci 3: 112. 10.3389/fpls.2012.00310 PMC3555118 23355841

[14] Lok N, Ho CM, Chen R, He QY, Yu WY, et al (2006) Proteomic Analysis of the Mode of Antibacterial Action of Silver Nanoparticles. J Proteome Res 5: 916–924. 16602699 10.1021/pr0504079

[15] Gao Y, Gopee NV, Howard PC, Yu L-R (2011) Proteomic analysis of early response lymph node proteinsin mice treated with titanium dioxide nanoparticles. Proteomics 74: 2745–2759. 21884834 10.1016/j.jprot.2011.08.009 PMC3215788

[16] Jeon YM, Park SKi, Lee MY (2011) Proteomic analysis of hepatotoxicity induced by titanium nanoparticles in mouse liver. J Korean Soc Appl Biol Chem 54: 852–859.

[17] Yang X, Liu J, He H, Zhou L, Gong C, et al (2010) SiO2 nanoparticles induce cytotoxicity and protein expression alteration in HaCaTcells. Part Fibre Toxicol 7(1): 1–9. 20180970 10.1186/1743-8977-7-1 PMC2830991

[18] Marsoni M, Bracale M, Espen L, Prinsi B, Negri AS, et al (2008) Proteomic analysis of somatic embryogenesis in *Vitis vinifera* . Plant Cell Rep 27 347–356. 17874111 10.1007/s00299-007-0438-0

[19] Marsoni M, Cantara C, De Pinto MC, Gadaleta C, De Gara L, et al (2010) Exploring the soluble proteome of Tobacco Bright Yellow-2 cells at the switch towards different cell fates in response to heat shocks. Plant Cell Environ 33: 1161–1175. 20199619 10.1111/j.1365-3040.2010.02137.x

[20] Mackey AJ, Haystead TA, Pearson WR (2002) Getting more from less: algorithms for rapid protein identification with multiple short peptide sequences. Mol Cell Proteomics 1: 139–147. 12096132 10.1074/mcp.m100004-mcp200

[21] Lin D, Xing B (2007) Phytotoxicity of nanoparticles: Inhibition of seed germination and root growth. Environ Pollut 150: 243–250. 17374428 10.1016/j.envpol.2007.01.016

[22] Yin L, Cheng Y, Espinasse B, Colman BP, Auffan M, et al (2011) More than the Ions: The Effects of *Silver Nanoparticl*es on *Lolium multiflorum.* . Environ Sci Technol 45: 2360–2367. 21341685 10.1021/es103995x

[23] Kim S, Lee S, Lee I (2012) Alteration of Phytotoxicity and Oxidant Stress Potential by Metal Oxide Nanoparticles in *Cucumis sativus.* Water Air Soil Pollution, DOI 10.1007/s11270-011-1067-3

[24] Griffitt RJ, Hyndman K, Denslow ND, Barber DS (2009) Comparison of molecular and histological changes in zebrafish gills exposed to metallic nanoparticles. Toxicol Sci 107: 404–415. 19073994 10.1093/toxsci/kfn256

[25] Domingos RF, Simon DF, Hauser C, Wilkinson KJ (2011) Bioaccumulation and Effects of CdTe/CdS Quantum Dots on Chlamydomonas reinhardtii – Nanoparticles or the Free Ions? Environ Sci Technol 45: 7664–7669. 21842898 10.1021/es201193s

[26] Kissen R, Bones AM (2009) Nitrile-specifier proteins involved in glucosinolate hydrolysis in Arabidopsis thaliana. J Biol Chem 284: 12057–12070. 19224919 10.1074/jbc.M807500200 PMC2673275

[27] Mithen RF (2001) Glucosinolates and their degradation products. In Advances in Botanical Research, Academic Press, 35, 213–262.

[28] Nikiforova V, Freitag J, Kempa S, Adamik M, Hesse H, et al (2003) Transcriptome analysis of sulfur depletion in Arabidopsis thaliana: interlacing of biosynthetic pathways provides response specificity. Plant J 33: 633–650. 12609038 10.1046/j.1365-313x.2003.01657.x

[29] Steinitz B, Bilavendran AD (2011) Thiosulfate stimulates growth and alleviates silver and copper toxicity in tomato root cultures. Plant Cell Tissue Organ Culture 107: 355–363.

[30] Gondikas AP, Morris A, Reinsch BC, Marinakos SM, Lowry GV, et al (2012) Cysteine-induced modifications of zero-valent silver nanomaterials: implications for particle surface chemistry, aggregation, dissolution, and silver speciation. Environ Sci Technol 46: 7037–7045. 22448900 10.1021/es3001757

[31] Duressa D, Soliman K, Taylor R, Senwo Z (2011) Proteomic analysis of soybean roots under aluminum stress. Int J Plant Genomics. doi:10.1155/2011/282531 10.1155/2011/282531 PMC3092509 21577316

[32] Xu XM, Lin H, Maple J, Björkblom B, Alves G, et al (2010) The Arabidopsis DJ-1a protein confers stress protection through cytosolic SOD activation. J Cell Sci 123: 1644–1651. 20406884 10.1242/jcs.063222

[33] Sanjaya, Hsiao PY, Su RC, Ko SS, Tong CG, et al (2008) Overexpression of Arabidopsis thaliana tryptophan synthase beta 1 (AtTSB1) in Arabidopsis and tomato confers tolerance to cadmium stress. Plant Cell Environ 8: 1074–85. 10.1111/j.1365-3040.2008.01819.x 18419734

[34] Watanabe N, Lam E (2011) Arabidopsis metacaspase 2d is a positive mediator of cell death induced during biotic and abiotic stresses. Plant J 66: 969–982. 21395887 10.1111/j.1365-313X.2011.04554.x

[35] Oukarroum A, Barhoumi L, Pirastru L, Dewez D (2013) Silver nanoparticle toxicity effect on growth and cellular viability of the aquatic plant *Lemna gibba* Environ Toxicol Chem doi: 10.1002/etc.2131 10.1002/etc.2131 23341248

[36] Lim D, Roh J, Eom H, Choi JY, Hyun JW, et al (2012) Oxidative stress-related PMK-1 P38 MAPK activation as a mechanism for toxicity of silver nanoparticles to reproduction in the nematode *Caenorhabditis elegans* . Environ Toxicol Chem 31: 585–592. 22128035 10.1002/etc.1706

[37] Asharani PV, Lian WY, Gong Z, Valiyaveettil S (2008) Toxicity of silver nanoparticles in zebrafish models. Nanotechnology 19: 1–8. 21828644 10.1088/0957-4484/19/25/255102

[38] Zhang R, Piao Mj, Kim KC, Kim AD, Choi JY, et al (2012) Endoplasmic reticulum stress signaling is involved in silver nanoparticles-induced apoptosis. Int J Biochem Cell Biol 44: 224–232. 22064246 10.1016/j.biocel.2011.10.019

[39] Bouwmeester H, Poortman J, Peters RJ, Wijma E, Kramer E, et al (2011) Characterization of translocation of silver nanoparticles and effects on whole-genome gene expression using an in vitro intestinal epithelium coculture model. ACS Nano 5: 4091–4103. 21480625 10.1021/nn2007145

[40] Christen V, Fent K (2012) Silica nanoparticles and silver-doped silica nanoparticles induce endoplasmatic reticulum stress response and alter cytochrome P4501A activity. Chemosphere 87: 423–434. 22245057 10.1016/j.chemosphere.2011.12.046

