# Supplementary figures and images for: Morphological and Proteomic Responses of Eruca sativa Exposed to Silver Nanoparticles or Silver Nitrate
Source: PLoS One. 2013 Jul 18;8(7):e68752. doi: 10.1371/journal.pone.0068752 (PMC3715538; doi:10.1371/journal.pone.0068752)

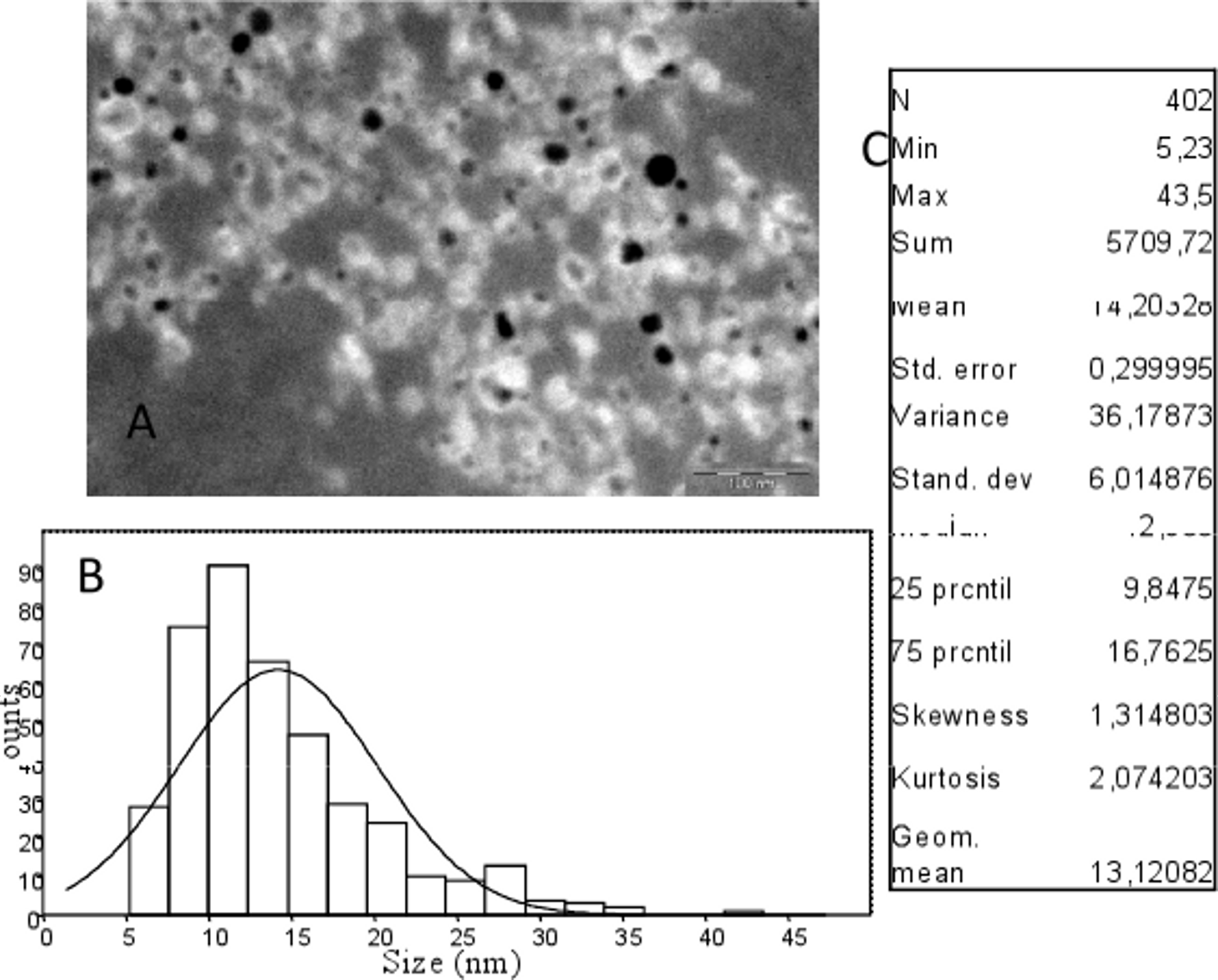

Supplement: Figure S1 — Detailed characterization of AgNPs. A: representative TEM image of 10 mg L−1 AgNp suspension. Magnification bar = 100 nm. B and C: size distribution of AgNPs. (TIF) [file pone.0068752.s001.tif]
