# Supplementary material for: Morphological and Proteomic Responses of Eruca sativa Exposed to Silver Nanoparticles or Silver Nitrate
Source: PLoS One. 2013 Jul 18;8(7):e68752. doi: 10.1371/journal.pone.0068752 (PMC3715538; doi:10.1371/journal.pone.0068752)
Supplement: Table S1 — Measurement of Ag content by F-AAS. (DOC) [file pone.0068752.s004.doc]

| Table S1. Measurement of Ag content by F-AAS. | |
| --- | --- |
| SAMPLE | Ag concentration measured ( ± SD) |
| 10 mg L^-1^ 10 nm AgNP suspension | 10.7 ± 1.7 mg L^-1^ |
| Ag content in AgNP treated roots | 8.3 ± 0.9 ng mg^-1^DW |
| Ag content in AgNP treated shoots | 0.014 ± 0.006 ng mg^-1^DW |
| Ag content in AgNO_3_ treated roots | 6.7 ± 0.6 ng mg^-1^DW |
| Ag content in AgNO_3_ treated shoots | 0.008 ± 0.0061 ng mg^-1^DW |
| DW= Dry Weight | |
