# Supplementary material for: Morphological and Proteomic Responses of Eruca sativa Exposed to Silver Nanoparticles or Silver Nitrate
Source: PLoS One. 2013 Jul 18;8(7):e68752. doi: 10.1371/journal.pone.0068752 (PMC3715538; doi:10.1371/journal.pone.0068752)
Supplement: Table S2 — Differentially expressed proteins in samples treated with AgNPs with respect to the control identified by CHIP-q-TOF MS/MS analysis. (DOC) [file pone.0068752.s005.doc]

| **Table S2. Differentially espressed proteins in samples treated with AgNPs with respect to the control identified by CHIP-q-TOF MS/MS analysis.** | | | | | | | | | |
| --- | --- | --- | --- | --- | --- | --- | --- | --- | --- |
| **Spot** | **Acc. N.** | **Species** | **Protein description** | **pI/MW Exp.** | **pI/MW Theor.** | **%**  **Cov.** | **Pep** | **Fold** | **Anova** |
| **Metabolism** | | | | | | | | | |
| 898 | O50008.1 | *Arabidopsis thaliana* | Vitamin-B12-independent methionine synthase | 6.0 / 72.0 | 6.0 / 84.4 | 7 | 4 (5) | +1.9 | 0.02 |
| 1944 | Q9SR37 | *Arabidopsis thaliana* | Beta-glucosidase 23 ^b^ | 6.5 / 39.0 | 6.3 / 57.2 ^b^ | 8 ^b^ | 3 (4) | +2.4 | 0.08 |
| 2068 | P93819 | *Arabidopsis thaliana* | Malate dehydrogenase, cytoplasmic 1 | 6.5 / 35.0 | 6.1 / 35.6 | 14 | 4 (5) | +1.5 | 0.015 |
| 3648 | O23733.1 | *Brassica juncea* | Cysteine synthase | 5.4 / 32.0 | 5.7 / 33.9 | 17 | 4 (4) | +2.3 | 0.037 |
| **Cell cycle** | | | | | | | | | |
| 2998 | Q944W6.1 | *Brassica oleracea* | Translationally-controlled tumor protein homolog | 4.6 / 18.0 | 4.6 / 19.0 | 34 | 5 (6) | -1.7 | 0.01 |
| **Defence / Stress related proteins** | | | | | | | | | |
| 3638 | XP_002862555.1 | *Arabidopsis lyrata* | Jacalin lectin family protein | 6.0 / 51.0 | 5.6 / 51.3 | 8 | 3 (3) | +3 | 0.003 |
| 2890 | AAC15842.1 | *Raphanus sativus* | Superoxide dismutase | 6.2 / 19.0 | 6.0 / 23.8 | 12 | 2 (3) | +1.5 | 0.026 |
| 3382 | AAD33602.1 | *Brassica rapa* | Type 2 peroxiredoxin | 5.5 / 12.0 | 5.4 / 17.4 | 36 | 4 (4) | +1.6 | 0.04 |
| 2574 | Q39366.1 | *Brassica oleracea* | Lactoylglutathione lyase-like protein | 5.6 / 27.0 | 5.2 / 39.3 | 10 | 2 (3) | +2 | 0.04 |
| **Protein fate** | | | | | | | | | |
| 3651 | Q9LKR3.1 | *Arabidopsis thaliana* | Luminal-binding protein 1 ^a^ | 5.1 / 69.0 | 5.1 / 73.6 | 12 | 5 (7) | -1.9 | 0.02 |
| 3625^d^ | XP_002868566.1 | *Arabidopsis lyrata* | Luminal-binding protein-2 ^a^ | 5.1 / 60.0 | 5.1 / 73.6 | 20 | 11(12) | -1.6 | 0.02 |
|  | Q38950.2 | *Arabidopsis thaliana* | Ser/thr-protein phosphatase 2A 65 kDa regulatory sub A beta isoform |  | 5.0 / 65.6 | 19 | 8 (11) |  |  |
| 3158 | P19036.2 | *Arabidopsis thaliana* | 17.4 kDa class I heat shock protein | 5.5 / 16.0 | 5.2 / 17.4 | 11 | 2 (2) | +2.2 | 2.4 e^-4^ |
| 3630 | Q9LTX9.1 | *Arabidopsis thaliana* | Heat shock protein 70-2 | 4.7 / 70.0 | 5.2 / 77.0 | 18 | 8 (10) | -1.8 | 0.03 |
| 3640 | P22953.3 | *Arabidopsis thaliana* | Heat shock cognate 70 kDa protein 1 | 5.1 / 66.0 | 5.0 / 71.4 | 38 | 18(24) | -2.1 | 0.014 |
| **Transport** | | | | | | | | | |
| 1901 | EFH66232.1 | *Arabidopsis lyrata* | V-type proton ATPase subunit C ^a^ | 5.3 / 40.0 | 5.4 / 43.0 | 27 | 9 (12) | -1.5 | 0.04 |
| 3691 | Q40079 | *Hordeum vulgare* | V-type proton ATPase subunit B2 | 5.0 / 52.0 | 5.1 / 53.7 | 31 | 8 (11) | -1.6 | 0.02 |
| **Reserve** | | | | | | | | | |
| 3654 | CAA42477.1 | *Raphanus sativus* | Cruciferin | 6.0 / 17.0 | 8.6 / 25.9 ^c^ | 31 ^c^ | 4 (4) | +2.1 | 0.006 |
| 3189 | CAA42477.1 | *Raphanus sativus* | Cruciferin | 5.5 / 16.0 | 8.6 / 25.9 ^c^ | 18 ^c^ | 2 (2) | +2.1 | 0.03 |
| 3633 | CAA42477.1 | *Raphanus sativus* | Cruciferin | 6.0 / 18.0 | 8.6 / 25.9 ^c^ | 23^c^ | 3 (3) | +1.7 | 0.02 |
| 3660 | CAA42477.1 | *Raphanus sativus* | Cruciferin | 6.0 / 18.0 | 8.6 / 25.9 ^c^ | 23 ^c^ | 3 (4) | +2.2 | 0.006 |
| 3071 | P11090.1 | *Brassica napus* | Cruciferin – subunit beta ^b^ | 6.7 / 18.0 | 6.2/ 20.8 ^b^ | 32 ^b^ | 4 (4) | +6.7 | 0.001 |
| 2979 | P11090.1 | *Brassica napus* | Cruciferin –subunit beta ^b^ | 6.6 / 18.0 | 6.2 / 20.8 ^b^ | 37 ^b^ | 4 (4) | +3.2 | 0.002 |
| a) Sequence annotation derived from BLAST alignment against nr-NCBI database. b) Values referred to the mature form of the protein. c) Partial sequence (fragment). d) Possible electrophoretic comigration. | | | | | | | | | |
