# Supplementary material for: Morphological and Proteomic Responses of Eruca sativa Exposed to Silver Nanoparticles or Silver Nitrate
Source: PLoS One. 2013 Jul 18;8(7):e68752. doi: 10.1371/journal.pone.0068752 (PMC3715538; doi:10.1371/journal.pone.0068752)
Supplement: Table S3 — Differentially espressed proteins in samples treated with AgNO3 with respect to the control identified by CHIP-q-TOF MS/MS analysis. (DOC) [file pone.0068752.s006.doc]

| **Table S3. Differentially espressed proteins in samples treated with AgNO_3_ with respect to the control identified by CHIP-q-TOF MS/MS analysis.** | | | | | | | | | | |
| --- | --- | --- | --- | --- | --- | --- | --- | --- | --- | --- |
| **Spot** | **Acc. N.** | **Species** | **Protein description** | **pI/MW Exp.** | | **pI/MW Theor.** | **%**  **Cov** | **Pep** | **Fold** | **Anova** |
| **Metabolism** | | | | | | | | | | |
| 1160 | NP_196728.1 | *Arabidopsis thaliana* | Malate dehydrogenase (NADP+) | 5.5 / 64.0 | | 6.0 / 64.4 | 25 | 13 (15) | +1.7 | 0.001 |
| 1323 | P56757.1 | *Arabidopsis thaliana* | ATP synthase subunit alpha, chloroplastic | 5.2 / 58.0 | | 5.2 / 55.3 | 13 | 5 (5) | +1.7 | 0.006 |
| 1338 | P56757.1 | *Arabidopsis thaliana* | ATP synthase subunit alpha, chloroplastic | 5.3 / 58.0 | | 5.2 / 55.3 | 22 | 9 (9) | +1.6 | 0.002 |
| 3545 | A4QK25.1 | *Arabis hirsuta* | ATP synthase subunit beta, chloroplastic | 5.4 / 52.0 | | 5.4 / 53.9 | 63 | 22 (27) | +2.3 | 7.4e-005 |
| 1403 | Q9C5A9.1 | *Arabidopsis thaliana* | ATP synthase subunit beta-3, mitochondrial | 5.4 / 55.0 | | 5.4 / 54.3 ^b^ | 29 ^b^ | 12 (13) | +1.7 | 0.006 |
| 3562 | P83484.1 | *Arabidopsis thaliana* | ATP synthase subunit beta-2, mitochondrial | 5.3 / 56.0 | | 5.4 / 54.2 ^b^ | 24 ^b^ | 10 (10) | +1.8 | 6e-004 |
| 3584 | Q9SJ12.1 | *Arabidopsis thaliana* | ATP synthase 24 kDa subunit, mitochondrial | 6.3 / 25.0 | | 5.3 / 24.0 ^b^ | 24 ^b^ | 5 (7) | +1.6 | 0.01 |
| 2679 | Q9SJ12.1 | *Arabidopsis thaliana* | ATP synthase 24 kDa sub., mitochondrial | 6.1 / 25.0 | | 5.3 / 24.0 ^b^ | 27 ^b^ | 5 (9) | +1.7 | 0.02 |
| 2642 | AAP82017.1 | *Brassica oleracea* | Tryptophan synthase alpha chain, partial | 5.2 / 26.0 | | 5.1 / 24.0 | 9 | 3 (3) | +2.0 | 0.001 |
| 1862 | AAP96742.1 | *Brassica rapa* | Class I glutamine amidotransferase ^a^ | 5.5 / 42.0 | | 5.3 / 41.7 | 41 | 13 (25) | +2.4 | 0.003 |
| 2330 | ACX70136.1 | *Brassica rapa* | O-acetylserine(thiol)lyase isoform A6 | 5.7 / 32.0 | | 5.7 / 33.9 | 37 | 9 (19) | +5.7 | 0.009 |
| 1954 | NP_173047.1 | *Arabidopsis thaliana* | Cinnamoyl CoA reductase 1 | 6.3 / 40.0 | | 6.1 / 37.5 | 26 | 8 (9) | +2.0 | 0.03 |
| 2546 | Q9C6B3.1 | *Arabidopsis thaliana* | Gamma carbonic anhydrase 2, mitochondrial | 6.0 / 28.0 | | 6.7 / 30.1 | 36 | 9 (11) | +2.1 | 0.04 |
| 3594 | XP_002883704.1 | *Arabidopsis lyrata* | Aconitate hydratase 2, mitochondrial ^a^ | 6.0 / 87.0 | | 6.6 / 108.5 | 12 | 9 (12) | +2.6 | 0.02 |
| 283 | O64517.1 | *Arabidopsis thaliana* | Metacaspase-4 subunit p20 | 4.7 / 21.0 | | 4.5 / 24.9 ^b^ | 8 ^b^ | 2 (3) | -2.3 | 2.5e-004 |
| **Protein synthesis** | | | | | | | | | | |
| 646 | BAJ33766.1 | *Thellungiella halophila* | Elongation factor EF-2 ^a^ | 6.5 / 88.0 | | 5.9 / 93.9 | 26 | 19(20) | -2.8 | 0.003 |
| 3563 | EFH70813.1 | *Arabidopsis lyrata* | Elongation factor EF-2 ^a^ | 6.4 / 88.0 | | 5.8 / 94.0 | 6 | 4 (5) | -2.6 | 9e-004 |
| 1735 | XP_002869935.1 | *Arabidopsis lyrata* | Chloroplast elongation factor tub | 5.5 / 45.0 | | 5.8 / 51.6 | 36 | 13(16) | +2.0 | 8e-004 |
| 3276 | AAR91929.1 | *Brassica napus* | Eukaryotic translation initiation factor-5A | 5.5 / 13.0 | | 5.7 / 17.1 | 37 | 4 (4) | +1.8 | 0.001 |
| **Defence / Stress related proteins** | | | | | | | | | | |
| 2784 | Q9ZRW8.1 | *Arabidopsis thaliana* | Glutathione S-transferase U19 | 5.6 / 22.0 | | 5.8 / 25.6 | 26 | 10 (12) | +1.8 | 0.003 |
| 2791 | Q9ZRW8.1 | *Arabidopsis thaliana* | Glutathione S-transferase U19 | 5.8 / 22.0 | | 5.8 / 25.6 | 21 | 7 (8) | +1.5 | 0.008 |
| 2846 | XP_002880321.1 | *Arabidopsis lyrata* | Glutathione S-transferase | 5.7 / 21.0 | | 6.3 / 24.1 | 21 | 5 (5) | +2.0 | 0.006 |
| 2856 | Q9FUS6.1 | *Arabidopsis thaliana* | Glutathione S-transferase U13 | 5.5 / 21.0 | | 5.6 / 25.1 | 5 | 2 (3) | +1.7 | 0.006 |
| 2888 | BAJ34627.1 | *Thellungiella halophila* | Glutathione S-transferase 11 ^a^ | 6.1 / 20.0 | | 5.7 / 23.7 | 47 | 12 (26) | +2.4 | 0.003 |
| 2933 | AAC15842.1 | *Raphanus sativus* | Superoxide dismutase | 6.2 / 19.0 | | 6.0 / 23.8 | 19 | 4 (6) | +4.6 | 0.002 |
| 2278 | BAJ34425.1 | *Thellungiella halophila* | Isoflavone reductase P3 ^a^ | 5.6 / 33.0 | | 5.4 / 34.1 | 16 | 5 (5) | +2.4 | 0.02 |
| 3308 | AAD33602.1 | *Brassica rapa* | Type 2 peroxiredoxin | 5.5 / 12.0 | | 5.4 / 17.4 | 26 | 3 (3) | +2.0 | 0.002 |
| 3404 | XP_002867694.1 | *Arabidopsis lyrata* | Major latex-related protein ^a^ | 5.3 / 11.0 | | 5.6/ 17.6 | 12 | 2 (3) | +4.5 | 9.5e-004 |
| 3582 | XP_002867694.1 | *Arabidopsis lyrata* | Major latex-related protein ^a^ | 6.0 / 11.0 | 5.6 / 17.6 | | 28 | 5 (8) | +2.3 | 0.001 |
| 3410 | ACC91258.1 | *Capsella rubella* | Major latex-related protein | 6.2 / 11.0 | 5.7 / 17.6 | | 25 | 4 (5) | +3.3 | 0.002 |
| 3344 | Q9ZVF3.1 | *Arabidopsis thaliana* | MLP-like protein 328 | 6.0 / 12.0 | 5.4 / 17.5 | | 10 | 2 (4) | +3.1 | 0.003 |
| 3423 | Q9ZVF3.1 | *Arabidopsis thaliana* | MLP-like protein 328 | 6.3 / 11.0 | 5.4 / 17.5 | | 28 | 7 (7) | +6.3 | 1.6e-004 |
| 3446 | Q9ZVF3.1 | *Arabidopsis thaliana* | MLP-like protein 328 | 6.2 / 11.0 | 5.4 / 17.5 | | 17 | 2 (2) | +1.8 | 0.01 |
| 3351 | Q9ZVF3.1 | *Arabidopsis thaliana* | MLP-like protein 328 | 5.7 / 11.0 | 5.4 / 17.5 | | 12 | 2 (3) | +3.1 | 0.005 |
| 2611 | NP_565003.3 | *Arabidopsis thaliana* | MLP-like protein 34 | 5.2 / 27.0 | 5.4 / 26.2 | | 19 | 6 (6) | +4.0 | 0.002 |
| 3553 | EFH54210.1 | *Arabidopsis lyrata* | Universal stress protein family protein | 6.4 / 11.0 | 5.5 / 18.1 | | 30 | 5 (7) | +2.1 | 0.005 |
| 3573 | EFH59266.1 | *Arabidopsis lyrata* | Jacalin-related lectin 31 ^a^ | 6.1 / 29.0 | 5.3 / 32.0 | | 9 | 4 (6) | +2.3 | 0.007 |
| 2496 | EFH59266.1 | *Arabidopsis lyrata* | Jacalin-related lectin 31 ^a^ | 6.0 / 29.0 | 5.3 / 32.0 | | 6 | 2 (4) | +2.5 | 0.008 |
| 3565 | XP_002862555.1 | *Arabidopsis lyrata* | Jacalin lectin family protein | 6.2 / 52.0 | 5.6 / 51.3 | | 15 | 8 (14) | +2.4 | 0.002 |
| 3567 | XP_002862555.1 | *Arabidopsis lyrata* | Jacalin lectin family protein | 6.0 / 51.0 | 5.6 / 51.3 | | 18 | 9 (15) | +3.3 | 0.002 |
| 1199 | BAB17227.1 | *Raphanus sativus* | Myrosinase | 6.4 / 63.0 | 7.1 / 62.2 | | 16 | 8 (9) | +1.7 | 0.04 |
| 1843 | Q42546.1 | *Arabidopsis thaliana* | SAL1 phosphatase | 5.2 / 42.0 | 5.0 / 37.6 | | 6 | 2 (2) | +2.1 | 0.006 |
| **Reserve** | | | | | | | | | | |
| 3089 | P11090.1 | *Brassica napus* | Cruciferin, beta subunit | 6.0 / 17.0 | 6.2 / 20.8 ^b^ | | 15 ^b^ | 2 (2) | -2.6 | 0.002 |

Detailed information about statistical data and spectra interpretation are reported in supplemental material. a) Sequence annotation derived from BLAST alignment against nr-NCBI database. b) Values referred to the mature form of the protein.
