# Supplementary material for: Morphological and Proteomic Responses of Eruca sativa Exposed to Silver Nanoparticles or Silver Nitrate
Source: PLoS One. 2013 Jul 18;8(7):e68752. doi: 10.1371/journal.pone.0068752 (PMC3715538; doi:10.1371/journal.pone.0068752)
Supplement: Methods S1 — NanoLiquid Chromatography-nanoElectroSpray Ionization-tandem mass spectrometry (nLC-nESI-MS/MS). (DOC) [file pone.0068752.s008.doc]

**Methods S1. NanoLiquid Chromatography-nanoElectroSpray Ionization-tandem mass spectrometry (nLC-nESI-MS/MS).** The extracted tryptic peptides were analyzed on an Agilent 6520 Q-TOF mass spectrometer with an HPLC Chip Cube source (Agilent Technologies). The chip consisted of a 40-nL enrichment column (Zorbax 300SB-C18, 5 µm pore size) and a 43-mm separation column (Zorbax 300SB-C18, 5 µm pore size) driven by the Agilent Technologies 1200 series nano/capillary LC system. Both systems were controlled by MassHunter Workstation Acquisition (version B.02.01, B2116.20; Agilent Technologies). Peptides were loaded onto the trapping column at 4 µL min^-1^ in 5% (v/v) acetonitrile and 0.1% (v/v) formic acid. The chip was then switched to separation, and peptides were eluted into the mass spectrometer during a 13-min acetonitrile gradient (from 5% to 60% v/v) in 0.1% (v/v) formic acid at 0.4 µl min^-1^. The mass spectrometer ran in positive ion mode and MS scans were acquired over a range from 300 to 3000 mass-to-charge ratio at 4 spectra s^-1^. Precursor ions were selected for auto-MS/MS at an absolute threshold of 1000 and a relative threshold of 0.01%, with a maximum of 4 precursors per cycle and active exclusion set at 2 spectra and released after 0.1 min. Analysis of MS/MS spectra for peptides identification was performed by protein database searching with Spectrum Mill MS Proteomics Workbench (Rev A.03.03.084; Agilent Technologies). Raw spectra were extracted to MS/MS spectra accepting a minimum sequence length of 3 amino acids and merging scans with the same precursor within a mass window of ±0.4 mass-to-charge ratio in a time frame of ±15 s. Charges up to a maximum of 7 were assigned to the precursor ion and the ^12^C peak was determined by Data Extractor. Key search parameters were Scored Peak Intensity (SPI) ≥ 50%, precursor mass tolerance of ± 20 ppm and product mass tolerance of ± 40 ppm. Carbamidomethylation of cysteines was set as fixed modification while variable modification was oxidation of methionines. Trypsin was selected as enzyme for sample digestion accepting 2 missed cleavages *per* peptide. The search was conducted against the subset of *Brassicaceae* protein sequences (Oct 2011, *331417 entries*) downloaded from the National Center for Biotechnology Information (NCBI).^1^ The database was concatenated with the reverse one. The threshold used for peptide identification was Spectrum Mill score ≥ 9, SPI% ≥ 50% and the difference between forward and reverse scores ≥ 2. If needed, identified peptides were used in protein similarity search performed by alignment analyses against the NCBI-nr database using the FASTS algorithm.^2^ Physical properties of the characterized proteins

S2

were predicted by in silico tools at ExPASy .^3^

1. National Center for Biotechnology Information [[http://www.ncbi.nlm.nih.gov](http://www.ncbi.nlm.nih.gov/)]
2. Mackey, A. J; Haystead, T. A. J; Pearson, W. R.Getting more from less: algorithms for rapid protein identification with multiple short peptide sequences**.** *Mol Cell Proteomics* **2002**, 1**,** 139-147.
3. **ExPASy Proteomics Server** [<http://www.expasy.org/>]
